# Effects of two types of numerical problems on the emotions experienced in adults and in 9-year-old children

**DOI:** 10.1371/journal.pone.0289027

**Published:** 2023-11-29

**Authors:** Maria Chiara Liverani, Eleni Kalogirou, Catherine Rivier, Edouard Gentaz

**Affiliations:** 1 Department of Psychology, Faculty of Psychology and Educational Sciences, University of Geneva, Geneva, Switzerland; 2 Department of Educational Sciences, Faculty of Psychology and Educational Sciences, University of Geneva, Geneva, Switzerland; 3 Swiss Center for Affective Sciences, University of Geneva, Geneva, Switzerland; Ahvaz Jundishapur University: Ahvaz Jondishapour University of Medical Sciences, ISLAMIC REPUBLIC OF IRAN

## Abstract

It is widely acknowledged that emotions and cognition are closely related, and that negative emotions are detrimental on school achievement, especially on mathematical performance. On the other hand, positive emotions have a positive impact on motivation and cognitive abilities underlying the learning processes. Nevertheless, studies about the effects of experienced emotions on problem solving, a specific type of mathematical activity, are sparse. The present research focuses on experienced epistemic and achievement emotions after the resolution of two types of numerical word problems: the *application problems*, that requires the use of a specific and expected algorithm to be solved and are regularly proposed at school; and the *non-application problems*, which cannot be solved directly but using different solving strategies. This type of numerical word problems appears less frequently in French school curricula. In experiment 1, 105 adults (M = 24.4 years), of which the majority was university students, were involved in an online experiment with APs and NAPs problems and were asked to rate their experienced emotions after the resolution of the problems. In experiment 2, 65 children aged 9-year-old were asked to individually solve APs and NAPs problems with age-appropriate difficulty and then rate their associated emotions. The adults’ sample reported higher epistemic and achievement positive emotions towards APs compared to NAPs. In both adults and children NAPs were more associated to surprise than APs. In children anxiety was more experienced after resolution of NAPs than APs. Results suggest the importance of varying the types of problems proposed in school curricula so that children become accustomed to using different solving strategies. This approach could be useful in decreasing negative emotions toward mathematics such as anxiety, which begins to settle as early as elementary school.

## Introduction

The concept of emotion is extremely complex and difficult to evaluate, since its components are subjective and not directly observables. For this reason, a multitude of definitions of emotions is presented in the literature. Nevertheless, many of these definitions seem to agree that emotion is best defined as a multicomponent concept [1]. Sander and colleagues propose five components of emotion, encompassing affective, cognitive, motivational, expressive and physiological processes [2]. According to Sander [3], an emotion is an “*event-focused*, *two-step*, *fast process consisting of 1) relevance-based emotion elicitation mechanisms that 2) shape a multiple emotional response (i*.*e*., *action tendency*, *automatic reaction*, *expression and feeling)”*(page 23). In the last decades research has extensively shown that emotion and cognition are two intertwined processes that constantly influence each other [4]. More recently, a growing body of research has focused on the role of the emotional experience in the school context and in the learning processes [5–7]. Studies have shown that emotions have an impact on students’ well-being at school, on their learning abilities, and on the possibility to achieve their academic goals [8–10]. Pereira and colleagues highlighted that the correlation between emotions capacities and academic performance is stronger in younger students at earlier levels of education [11]. In their meta-analysis, MacCann and colleagues [9] found that the ability to understand and manage emotions are active ingredients in the prediction of academic performance from primary school to university years. In addition, students with higher emotional abilities not only have better relationship with teachers and peers but are more capable to regulate negative emotions such as anxiety, boredom or disappointment, that can be associated to school performance. Thus, negative emotions can be a vector of failure and loss of trust if they are inappropriate, unregulated or ignored. On the other hand, positive emotions can represent a powerful catalyst for school well-being and academic success. Pekrun criticizes the fact that studies in the educational domain are mainly focused on anxiety, neglecting other emotions that are supposed to exert negative or positive influences on achievement and to impact the cognitive strategies employed in the learning process and motivation [12, 13]. According to Pekrun and colleagues [12] there is a large emotional repertoire linked to school, and they have proposed a categorization of the emotions experienced in the educational domain. All emotions, except disgust, are reported to be experienced as part of schooling. In line with this categorization, emotions that are directly related to the knowledge-generating qualities of cognitive tasks are defined as epistemic emotions, while emotions that are related to the outcome–academic success or failure–are defined as achievement emotions [14–16]. According to Muis et al. [17], epistemic emotions can be both positive and negative, and include—but are not limited to—curiosity, enjoyment, confusion, boredom and interest. They are all closely associated and play a key role in learning, knowledge acquisition and novelty exploration. Achievement emotions can also be positive and negative, such as enjoyment, boredom and anxiety. They influence performance, and performance influence achievement in a relation of reciprocal causation [18].

Based on this theoretical framework, the following research aimed to investigate the association between these two types of emotions (epistemic and achievement emotions) and mathematics, which is one of the school subjects often reported to be tightly linked with emotions. A large body of literature has focused on the impact of negative emotions on math performance, and the term “math anxiety” has been coined, defining an adverse emotional reaction to math or to the prospect of doing math [19]. The influence of anxiety on mathematical achievement is detrimental. The more anxious students are towards math, the less they succeed [20, 21]. The mechanism subjacent to this negative outcome may be that anxiety co-opts a portion of the limited working memory resources that should normally be allocated to successfully resolving a mathematical problem [19]. In addition, anxiety towards math is usually specific, not generalized to other domains–such as reading–and not explained by students’ broader trait anxiety [21]. Most studies show that math anxiety starts to negatively impact performance from middle school onwards [19, 22]. Concerning lower grades, results are less consistent. While some studies suggest that in early primary school math anxiety is not yet correlated to performance [23, 24] other studies expose opposite data. Wu and colleagues [25] showed that anxiety and math performance are already negatively correlated during the 2^nd^ and 3^rd^ grades. Similarly, Punaro and Reeve studied 9-year-old children and found that the group with the greatest worries about math showed the worst performance [26].

In the present study, we decided to focus on a particular mathematical activity, which is the resolution of numerical word problems. It is a complex and multi-faceted activity, requiring several mathematical and general cognitive abilities [27, 28]. Given the complexity and variety of the processes involved, there is an extensive literature in educational psychology and mathematics didactics about the different type of problems [29, 30]. In addition, a growing number of studies are focusing on comparing the different typologies of problems presented to students and their representativeness in teaching manuals. For example, Zhu and Fan [31] showed that American students have to solve about twice as many problems as Chinese students, and that there is a massive predominance for both countries of routine problems, traditional problems and closed problems (≥ 93%). The US textbooks contained considerably fewer step problems than the Chinese textbooks, but a higher proportion of problems related to real-life situations. Similarly, Vicente and colleagues [32] compared textbooks from Singapore and Spain, concluding that Singapore textbooks included more reasoning problems than Spanish textbooks, even though they included fewer step problems. This heterogeneous distribution could have consequences on pupils’ learning because they are not used to solve different types of problems and to work on arithmetic notions in all their dimensions. As a consequence, the activity of problem solving can be associated to a large panel of experienced emotions that may differ between individuals having different levels of practice and different ages.

To the best of our knowledge, there are no studies investigating how specific types of mathematical problems can elicit different experienced emotions and how this varies at different ages. Therefore, the goal of the current study is to explore the effects of two types of numerical problems on the emotion experienced in young adults and 9-year-old children. Recently, we proposed two types of numerical problems to children aged 6 to 9 in classroom: the application problems (AP) and the non-application problems (NAPs) [33]. APs can be defined as statements that engage children in an expected procedure, mobilizing and training a particular mathematical notion, in connection with the pedagogical progression (e.g., "In Lou’s library, there are 4 shelves. On each shelf, Lou stored 8 books. How many books does she have?"). This type of problem is often proposed on French textbooks to pupils in cycle 2, aged from 6 to 9 [34, 35].

On the other hand, NAPs are numerical word problems whose resolution is not directly accessible to the child through the application of an algorithm studied in class (e.g., "A farmer has chickens and rabbits. Looking at all his animals, he sees 8 heads and 28 legs. How many rabbits does he have?”). Since this type of numerical word problem cannot be solved with the application of a systematic and trained algorithm, children must develop a personal and original strategy and test its effectiveness. Importantly, NAP statements must be in a conceptual domain familiar to the child, so that they can easily engage in empirical resolutions with a trial-and-error approach. NAPs allow the teacher to show that there is strategic variability to solve a problem. Children are asked to explore different strategies to find the solution, assuming a posture of active learning. As Freeman and colleagues pointed out in their meta-analysis [36], this active learning approach seems to be beneficial to learning and elicit students’ commitment. Nevertheless, this type of problem is rarely proposed by teachers or in school manuals, probably because of its complex and time-consuming nature.

The present study is composed of two experiments and has two main objectives: 1- to investigate how the two types of numerical verbal problems (APs and NAPs) influence the experienced emotions; 2- to investigate if this influence is different between adults and 9-year-old-children. Since APs are more common in the school curricula and manuals [34] and require the mere application of a specific algorithm, we expect that they will be more associated to positive epistemic and achievement emotions compared to NAPs, which are associated to an active research of the solution and less proposed to students. Furthermore, since adults have been trained to solve a large number of APs during their academic career, we predict that the effects of problem types on the experienced emotions will be greater in adults than in children. This research aims to fill the gap in the literature about the affective correlates of mathematical problem-solving. In this way, experienced emotions felt toward this school activity can be considered with their proper value, in order to promote positive emotions and reduce the negative ones.

Experiment 1 was conducted online due to Covid-19 restrictions, among young adults, of which the majority was university students of the Faculty of Psychology and Education at the University of Geneva, Switzerland. Experiment 2 was conducted in the classrooms with 9-year-old children in public schools all over the Geneva area.

## Experiment 1: Adult students

### Method

#### Participants and design

Adult participants were recruited using a snowballing recruitment technique and by word of mouth. 105 participants replied to all the questions of the online experiment (7 had to be excluded as they did not). Participants’ mean age was 24.4 years (SD = 7.41), 88 were females and 17 were males. Most participants held a bachelor’s degree (44.8%), while 39% were high-school graduates. Another 11.4% held a master’s degree, 3.8% held an advanced degree and only one participant did not graduate high school. Data collection took place in 2021 using Qualtrics, an online survey tool (Qualtrics^XM^, Provo, UT), following the implementation of health regulations related to the Covid-19 pandemic. The online questionnaire was in French and was composed of a set of applicative and a set of non-applicative problems (AP and NAP, respectively) in a randomized order, so that each participant was first presented either with the applicative or with the non-applicative problems. Participants were randomly assigned to a feedback (FB) or no-feedback group (NFB), meaning that depending on their condition, they either received feedback after providing the problem’s answer, or immediately proceeded to the next problem without receiving any feedback. Once participants completed a set of problems (applicative or non-applicative) they were asked to rate their current affective experience on 5-point Likert scales.

Before starting the experiment, participants were asked to agree or disagree to participate in the study through a written online consent form. The study was approved by the Ethics Committee of the University of Geneva (ID: PSE.20201188.MM).

#### Measures

*Applicative (AP) and Non-Applicative Problems (NAP)*. Four applicative and two non-applicative problems were randomly presented to participants (cf. Table 1 to see the proposed problems). As NAPs can be solved using different approaches and can therefore require more time to find the solution, we decided to propose only two problems of this type, and four APs. After each problem, participants were asked to provide the answer and report whether they used a calculator or not. They were also asked to indicate to what extent they were certain about their reply on a 4-point Likert scale (1 = I found the solution and I am certain that it is the right one, 2 = I found a solution but I doubt that it is the right one, 3 = I found a solution but I am certain that it is not the right one, 4 = I did not find the solution). Participant replies to the problems, use of calculator and ratings of certainty were not included in the final analysis.

**Table 1:** Applicative and non-applicative problems proposed to adult students.

Applicative problems	Non-applicative problems
The moon makes one complete revolution around the Earth in about 27.3 days. How many complete revolutions around the Earth does the Moon make in one year?	A farmer has goats and ducks. Looking at all his animals, he sees 38 heads and 122 legs. How many goats are there? How many ducks are there?
The cruising speed of an Airbus A380 is 900 km per hour. How many kilometers will it cover at this speed in 2h15min?	Eight teams participate in a soccer game. Each team must meet all the other teams. How many matches will be played in this game?
Mrs. Secure has installed a rectangular pool that is 20 m long and 9 m wide. She must now place a protective fence 2.50 m from the edges of the pool, leaving a 90 cm opening for a gate. In meters, how long is the fence?	
Three brothers sold their harvest of chanterelles at the market. Andy had 6 kg, Lucas 7 kg and Alexis, the smallest, doesn’t know how many. The mushrooms were sold for 12€ per kilo and the sale brought 180€ to the three brothers. How many kilos of chanterelles did Alexis pick?	

*Five-point Likert scales on affective experience*. after completing each set of problems, respondents were asked to report to what extent they experienced multiple epistemic and achievement emotions on a 5-point Likert scales (1 = not at all, 2 = very little, 3 = moderately, 4 = a lot, 5 = extremely). Epistemic and achievement emotions were chosen on the basis of previous questionnaires of Pekrun and colleagues: the Achievement Emotions Questionnaire [15], the Achievement Emotions Questionnaire–Elementary School [14] and the Epistemically-Related Emotion Scales [37], which were adapted using an in-house translation in French.

The instructions for the epistemic emotions were the following: “While I was looking for the solution of this problem, I felt…”, while for the achievement emotions the instructions were: “When I have finished looking for the solution of this problem, I felt…”. Epistemic emotions included: joy, happiness, excitement, curiosity, interest, nervousness, anxiety, worry, frustration, irritation, dissatisfaction, astonishment, surprise, monotony, boredom, perplexity, confusion. Achievement emotions included: relief, pride, joy, optimism, shame, despair, anger, anxiety. Therefore, participants had to rate on the Likert scale a total of 25 emotions (17 epistemic and 8 achievement emotions).

#### Data analysis plan

Statistical analyses were performed using the Statistical Package for Social Sciences (SPSS, software version 26.0). Initially, to explore the structure of the data on affective experience and reduce the number of variables, a Principal Component Analysis (PCA) was conducted for each experimental condition (AP with FB, AP without FB, NAP with FB, NAP without FB) for epistemic (4 PCAs) and for achievement (4 PCAs) emotions. A single PCA could not be performed due to the repeated-measures design of our study. After having reduced the dimensionality of data on affective experience, a two-way mixed MANOVA was conducted to assess changes in the experience of epistemic and achievement emotions (dependent variables) as a function of the type of problem (AP, NAP) and feedback condition (Feedback, No Feedback).

## Results

Reported ratings were entered into 8 separate Principal Component Analyses (PCA) with Varimax rotation, one for each type of emotion, type of problem and feedback condition. Sphericity (Bartlett’s), linearity, and sampling adequacy (KMO) assumptions for all PCAs were met. Sample size for PCAs conducted on the ‘Feedback’ condition was N = 47, while PCAs conducted on the ‘No Feedback’ condition had a sample size of N = 58 (see supporting information for strong component factor loadings, S1–S8 Tables). Across the PCAs, it can be observed that several emotions repeatedly load into the same components. For epistemic emotions, joy, happiness, excitement, curiosity and interest load into the same component across all the PCAs. The same can be observed for nervousness, anxiety and worry, as well as for surprise and astonishment. Finally, frustration, irritation and dissatisfaction also load into the same component in the majority of PCAs. Achievement emotions seem to present a simpler organization across PCAs with relief, pride, joy and optimism loading into one component, while shame, despair, anger and anxiety load into another component in most PCAs.

Based on these observations and in order to reduce the number of dependent variables for the MANOVA, we created mean scores by combining the variables that tended to load into the same component and used them as dependent variables in the MANOVA (Table 2). In addition to the ones mentioned above, we decided to create two additional mean score variables by combining boredom with monotony (r ≳ .5) and confusion with perplexity (r > .5). Given the nature of our data (ordinal), it would have been more appropriate to use modes in order to compute new variables. Unfortunately, this was not possible as our data were multimodal. Nevertheless, means remain informative for the interpretation of the MANOVA results.

**Table 2:** Descriptive Statistics for dependent variables used in MANOVA in Experiment 1.

		AP		NAP	
		M	SD	M	SD
Positive Epistemic Emotions (joy, happiness, excitement, curiosity, interest)	FB	3.17	.93	2.93	.99
	NFB	2.98	.93	2.81	.82
Stressful Epistemic Emotions (nervousness, anxiety, worry)	FB	1.72	.93	1.77	.85
	NFB	1.83	.95	1.95	1.11
Negative Epistemic Emotions (frustration, irritation, dissatisfaction)	FB	1.74	.93	2.01	1.13
	NFB	1.72	.94	2.15	1.17
Surprise Epistemic Emotions (surprise, astonishment)	FB	1.86	.91	2.14	1.17
	NFB	1.90	.89	2.34	1.07
Monotonous Epistemic Emotions (boredom, monotony)	FB	1.81	.85	1.63	.89
	NFB	1.82	.86	1.84	.80
Complexity Epistemic Emotions (confusion, perplexity)	FB	1.84	.90	2.56	1.13
	NFB	2.03	.86	2.72	1.05
Positive Achievement Emotions (relief, pride, joy, optimism)	FB	3.04	.92	2.54	.83
	NFB	2.68	.90	2.44	.83
Negative Achievement Emotions (shame, despair, anger, anxiety)	FB	1.40	0.66	1.63	.79
	NFB	1.48	.68	1.64	.76

A two-way mixed MANOVA was conducted on 8 dependent variables with “Feedback” (FB/NFB) as a between-group independent variable and “Type of problem” (AP/NAP) as a within-group independent variable. Even though tests of univariate normality appeared to be significant, we decided to proceed with the analysis as the Shapiro-Wilk test is known to be biased by large sample sizes and because the inspection of residual plots did not yield any concerns. Furthermore, MANOVA is generally robust to multivariate normality violations when groups are of approximately equal size, as in this case. Results showed a main effect for type of problem (F(8, 96) = 7.724, p = .000, η_p_^2^  = .392). Univariate tests indicated higher mean ratings on positive epistemic emotions for applicative than non-applicative problems (F(1, 103) = 10.064, p = .002, η_p_^2^  = .089). Lower mean ratings for applicative than non-applicative were observed for negative epistemic emotions (F(1, 103) = 15.843, p = .000, η_p_^2^  = .133), for epistemic emotions of surprise (F(1, 103) = 12.949, p = .000, η_p_^2^  = .112), as well as for epistemic emotions of complexity (F(1, 103) = 50.593, p = .000, η_p_^2^  = .329). With regards to achievement emotions, higher mean ratings for applicative than non-applicative were observed for positive achievement emotions (F(1, 103) = 26.039, p = .000, η_p_^2^  = .202). Lower ratings for applicative than non-applicative were observed for negative achievement emotions (F(1, 103) = 9.798, p = .002, η_p_^2^  = .087). There was no main effect for feedback and no significant interactions.

## Experiment 2: 9-years-old children

### Method

#### Sample and design

Child participants were recruited with the help of the Department of Public Education of the Canton of Geneva. School principals were contacted by the Department and sent back a list of educators who were interested in participating in the study with their classes. Researchers contacted the teachers and asked them to distribute the written consent forms to pupils’ parents. Children were asked to return the consent forms, signed by their parents, before the date of the group testing. A total of 65 children aged between 8 and 10 years and frequenting either the 5^th^ (N = 35) or 6^th^ (N = 30) grade participated in the study. Our sample included families with heterogeneous socio-economic status. As for the adult sample, children were randomly assigned to the feedback and no-feedback conditions. All children were asked to solve a set of APs and a set of NAPs (adapted to their age) in a random order. Participants were tested in two times: once collectively (for approx. 1 hour and 30 minutes) and once individually (for approx. 45 minutes). Individual sessions followed approximately within a month after the collective session. During the collective session, children initially completed a task of verbal comprehension called É.co.s.se [38]. The É.co.s.se evaluates the syntactic/semantic comprehension of statements. It consists of 23 blocs of 4 items, for a total of 92 items of increasing difficulty. Each item comprises a statement and four images, of which one illustrates the statement and the rest serve as distractors. Blocks J to W (56 items) corresponded to the age range of our sample and were used in our study. The evaluation takes place in two times: first, the statement is read out loud by the experimenter. Then, by turning the page, the child needs to choose the image that illustrates the statement, while at the same time listening to the next statement. All participants had very high scores in this task, reaching a ceiling effect (mean score = 39.53 out of 56, SD = 1.24). Subsequently, they randomly replied to a set of either APs or NAPs. Finally, children rated their current emotional experience by replying to a series of 5-point Likert scales. During the individual session, participants initially solved a set of either APs or NAPs problems (depending on what set they had solved in the collective session) and then rated their emotional current experience by replying to a series of 5-point Likert scales.

This study was approved by the Ethics Committee of the University of Geneva (ID: PSE.20201188.MM).

#### Measures

*Applicative (AP) and Non-Applicative Problems (NAP)*. children were presented with three APs and two NAPs adapted to their school grade (cf. Table 3 to see the problems proposed to our children sample).

**Table 3:** Applicative and non-applicative problems proposed in 9-year-old children.

Applicative problems	Non-applicative problems
Sixty- seven cars are stored in a garage that already contains 39 cars. How many cars are there in the garage?	A farmer has goats and ducks. Looking at all his animals, he sees 10 heads and 34 legs. How many goats are there? How many ducks are there?
For the school party, Alex brings 5 packages of 12 buns. How many buns did he bring?	Six teams participate in a soccer game. Each team must meet all the other teams. How many matches will be played in this game?
Thirty students sign up for a karate class. After one month, 11 students drop out. How many children are left in this class?	

*Five-point Likert scales on affective experience*. after completing each set of problems, children were asked to report to what extent they experienced multiple epistemic and achievement emotions on a 5-point Likert scale (1 = not at all, 2 = very little, 3 = moderately, 4 = a lot, 5 = extremely). Epistemic and achievement emotions were chosen on the basis of previous questionnaires of Pekrun and colleagues: the Achievement Emotions Questionnaire [15], the Achievement Emotions Questionnaire–Elementary School [14] and the Epistemically-Related Emotion Scales [37], which were adapted using an in-house translation in French.

The instructions for the epistemic emotions were the following: “While I was looking for the solution of this problem, I felt…”, while for the achievement emotions the instructions were: “When I have finished looking for the solution of this problem, I felt…”. We used the results of the PCA and MANOVA analyses in the adult sample in order to reduce the number of items for epistemic and achievement emotions that were used with children. Epistemic emotions included: curiosity, boredom, confusion, surprise, anxiety, frustration, joy and irritation. Achievement emotions included: relief, pride, joy, optimism, shame, despair, anger, anxiety.

#### Data analysis plan

Statistical analyses were performed using the Statistical Package for Social Sciences (SPSS software version 26.0). Due to the small sample of our study, it was not possible to conduct PCA analyses in order to reduce the number of dependent variables. Therefore, and given that our sample size was adequate for the number of dependent variables, we decided to proceed with a two-way mixed MANOVA in order to assess changes in the experience of epistemic and achievement emotions (dependent variables) as a function of type of problem (AP, NAP) and feedback condition (feedback, no feedback).

## Results

Our dataset presented 24 missing completely at random (MCAR) values at the 5-point Likert scales on affective experience. Since the repeated measures procedure in SPSS completely excludes from the analysis cases that present even one missing value, our sample size would have been reduced by 21,54% (N = 14 participants). For that reason, we proceeded with an iterative Markov Chain Monte Carlo (MCMC) method to impute missing values (10 iterations). Mean ratings and standard deviations for original and post imputation data are presented in Table 4 for each variable.

**Table 4:** Descriptive statistics for original data and complete data after imputation in Experiment 2.

	Imputed Values	Original Data		Imputed Data	
		M	SD	M	SD
Epistemic Anxiety (AP)	1	1.70	1.178	1.68	1.182
Epistemic Frustration (AP)	2	1.97	1.332	2.04	1.396
Epistemic Joy (AP)	2	3.46	1.412	3.40	1.477
Achievement Optimism (AP)	1	3.94	1.271	3.93	1.262
Achievement Pride (AP)	1	4.00	1.333	3.94	1.423
Achievement Anger (AP)	2	1.30	.796	1.29	.789
Achievement Shame (AP)	1	1.86	1.207	1.90	1.245
Epistemic Boredom (NAP)	1	2.02	1.175	2.01	1.167
Epistemic Confusion (NAP)	1	2.23	1.035	2.23	1.027
Epistemic Anxiety (NAP)	1	1.75	1.054	1.75	1.046
Epistemic Frustration (NAP)	1	2.31	1.344	2.31	1.333
Epistemic Joy (NAP)	1	3.03	1.425	3.07	1.446
Epistemic Irritation (NAP)	1	1.44	.941	1.47	.971
Achievement Optimism (NAP)	1	3.61	1.410	3.63	1.406
Achievement Pride (NAP)	1	3.84	1.312	3.84	1.303
Achievement Anger (NAP)	1	1.22	.723	1.20	.742
Achievement Shame (NAP)	1	2.09	1.318	2.12	1.331
Achievement Despair (NAP)	2	1.49	1.134	1.48	1.141
Achievement Anxiety (NAP)	2	1.78	1.156	1.79	1.139

A two-way mixed MANOVA was conducted on 16 dependent variables (8 for epistemic and 8 for achievement emotions) with feedback (FB/NFB) as a between-group independent variable and type of problem (AP/NAP) as a within-group independent variable. Even though tests of univariate normality appeared to be significant, we decided to proceed with the analysis as inspection of residual plots did not yield any concerns. Furthermore, MANOVA is generally robust to multivariate normality violations when groups are of approximately equal size, as in this case (N_FB_ = 35, N_NFB_ = 30). Results showed a main effect for type of problem (F (16, 48) = 2.222, p = .017, η_p_^2^  = .425). Univariate tests indicated lower mean ratings on epistemic surprise for applicative (M = 1.72, SD = .960) than non-applicative (M = 2.15, SD = 1.383) problems (F (1, 63) = 6.426, p = .014, η_p_^2^  = .093). For achievement anxiety, univariate tests indicated lower mean ratings for applicative (M = 1.48, SD = .970) than non-applicative (M = 1.79, SD = 1.139) problems (F (1, 63) = 5.714, p = .020, η_p_^2^  = .083). There was no main effect for feedback, nor a significant interaction.

## Discussion

With the present study we wanted to investigate if two types of numerical verbal problems (APs and NAPs) were associated to different epistemic and achievement emotions in adult students and 9-year-old children in the Geneva area. The aim was to shed light on the emotional components of problem solving, given the acknowledged effect of emotions on mathematical performance and academic achievement.

In the first experiment we proposed APs and NAPs to young adults. Results on epistemic emotions experienced after problem resolution showed higher levels of positive emotions (e.g., joy, curiosity, interest) and lower levels of negative emotions (e.g., frustration, irritation, dissatisfaction) after the resolution of APs compared to NAPs problems. According to the literature, epistemic emotions are triggered by the cognitive characteristics of tasks and are therefore related to knowledge [37]. As discussed in the introduction, NAPs are very rarely offered in school curricula and require the application of a different reasoning than the “classical” problems. The use of a simple algorithm is not enough to find the solution, and the implementation of an original resolution approach (among several possible strategies) is needed to solve the problem. Therefore, individuals are confronted to new cognitive characteristics that are peculiar to NAPs problems. Consequently, they could be spurred to implement new reasoning strategies to find a solution, feeling curiosity and motivation for the new challenge they are faced with, or—on the contrary—feel demoralized and blocked in their search for solutions, and thus experience negative emotions. The present results tend toward the second hypothesis: when faced to the new and unfamiliar cognitive characteristics of the NAPs, our participants experience difficulties and, therefore, they associate the practice of solving this type of problems to negative epistemic emotions such as frustration and dissatisfaction. This interpretation is corroborated by results on specific clusters of epistemic emotions. NAPs were more associated to surprise and complexity. According to the PCA analysis, the “surprise cluster” included the emotions of surprise and astonishment, while the “complexity cluster” encompassed confusion and perplexity. According to Kang and colleagues [39], these kinds of emotions are associated to the presence of unexpected information and to cognitive incongruity, which is the case of NAPs problems. Because of this unexpected information, individuals could experience either curiosity and attempt to try to resolve it, or confusion if they encounter difficulties towards the incongruity and are not able to finally resolve the conflict [40]. Our participants seem to experience more confusion in response to this type of problem than curiosity, and this could explain why they report more negative than positive affects towards NAPs. The same appears to be true for achievement emotions, as APs are more associated to emotions like optimism, pride and joy, while NAPs seem to elicit affects such as shame, despair and anxiety. The control-value theory of achievement emotions [41] states that the types of achievement emotions individuals experience depend on their perception of control (including both control of the action and of the outcome) as well as their value appraisals, which are associated to motivation, personal goals and cognitive quality. Participants were used to dealing with application problems, which are more often proposed in school. This practice must have positively impacted their perception of control, enhancing positive achievement emotions toward this type of problem. On the contrary, the surprise accompanying the new cognitive challenge represented by NAPs must have led participants to experience negative emotions such as—among others—anxiety, which is the most prominent negative affect associated to mathematics. Finally, the presence or not of the feedback after the problem resolution did not show any impact on experienced emotions. One possible reason could be that our feedback consisted of the simple transcription of the correct answer, without any explanation of the requested procedure for arriving at the result. As Van der Kleij and colleagues pointed out in their meta-analysis [42], this kind of feedback is not effective on student’s performance. Therefore, we could argue that there is no influence on the experienced emotions associated to the act of problem solving either.

The second experiment evaluated the same achievement and epistemic emotions after the resolution of APs and NAPs problems in 9-year-old children. In this sample, NAPs were more associated to epistemic surprise than APs. This result parallels what we found in the adult sample, and, as in adults, no effect of the feedback was found. In addition, children experienced higher levels of stressful epistemic emotions such as anxiety and worry when confronted to NAPs than APs. As already mentioned in the introduction, a large body of research exists on the link between anxiety and mathematics, and this link seems to increase with age [43]. Nevertheless, it is still unclear when is the key period during which this association start to set up. Our results are in line with studies showing an association between math and anxiety already in 9-year-old children [25, 26]. In addition, we can suggest another interesting point: anxiety is mainly perceived when students are confronted with problems of which they are not used to, and they feel more comfortable when faced to “classic” problems. We can argue that as they are free to mobilize different strategies during the NAPs resolution, leaving them free to search which strategy is the correct one, it is too stressful for them. This observation leads us to emphasize the importance of exposing students from an early age to different types of problems such as NAPs, so that they become familiar and comfortable with different methods of reasoning and research of solution. In this perspective Rivier and Sander [35] have recently proposed an innovative training program to elementary school students in which different problem-solving skills were trained using verbal statements. In this training were presented both “prototypic” problems, which can be solved based on our intuition, and “non-prototypic” problems, which are contrary to our intuitions. Results indicate that the training had a beneficial impact on student’s capacity to solve both prototypic and non-prototypic mathematical problems. Therefore, it would seem possible to train children to solve non-application and application problems.

Some limitations deserve to be mentioned for this study. First of all, the first experiment has been done online, due to Covid-19 pandemic’s restrictions. As with every online study, we cannot be aware of the conditions under which the questionnaires were filled by student participants. Regarding the absence of feedback effect, an immediate explicit explanation of the right solution for each problem would probably have been more efficient. Finally, appropriate analyses assessing the reliability and structural validity of the scales used to evaluate the experienced emotions should be performed in a larger sample size, in order to guarantee the quality of our measures.

In conclusion, our study shows that two types of mathematical verbal problems are associated to different epistemic and achievement experienced emotions, with more positive feelings toward application problems, which are largely proposed in the school curricula, as opposed to non-application problems that, being less well known, elicited more negative emotions. All together, these results underline the importance of exposing children to different types of mathematical problems already in the first years of elementary school. As Liljedahl and Cai propose [44], more research is needed on the topic of problem solving in school curricula. On the basis of our results, we believe that future research should not only focus on the pedagogical aspects on this topic, but also on the emotional correlates of this mathematical activity. In addition, future research should implement specific trainings, investigating their impact on experienced positive and negative emotions, with a special focus on math anxiety. This should prevent or at least reduce the association of negative emotions with student’s experience of mathematics and therefore positively impact performance and academic success.

## Supporting information

S1 Data (XLSX)

S2 Data (XLSX)

S1 Table Strong component factor loading (≥ .7) for each regression for epistemic emotions (E)—applicative problems (AP)—feedback (FB). Percentages of explained variance for each component are presented in parentheses.
(DOCX)

S2 Table Strong component factor loading (≥ .7) for each regression for epistemic emotions (E)—applicative problems (AP)—no feedback (NFB). Percentages of explained variance for each component are presented in parentheses.
(DOCX)

S3 Table Strong component factor loading (≥ .7) for each regression for epistemic emotions (E)–non-applicative problems (NAP)—feedback (FB). Percentages of explained variance for each component are presented in parentheses.
(DOCX)

S4 Table Strong component factor loading (≥ .7) for each regression for epistemic emotions (E)–non-applicative problems (NAP)—no feedback (NFB). Percentages of explained variance for each component are presented in parentheses.
(DOCX)

S5 Table Strong component factor loading (≥ .7) for each regression for achievement emotions (A)—applicative problems (AP)—feedback (FB). Percentages of explained variance for each component are presented in parentheses.
(DOCX)

S6 Table Strong component factor loading (≥ .7) for each regression for achievement emotions (A)—applicative problems (AP)—no feedback (NFB). Percentages of explained variance for each component are presented in parentheses.
(DOCX)

S7 Table Strong component factor loading (≥ .7) for each regression for achievement emotions (A)–non-applicative problems (NAP)—feedback (FB). Percentages of explained variance for each component are presented in parentheses.
(DOCX)

S8 Table Strong component factor loading (≥ .7) for each regression for achievement emotions (A)–non-applicative problems (NAP)–no feedback (NFB). Percentages of explained variance for each component are presented in parentheses.
(DOCX)
