# Supplementary material for: Effects of two types of numerical problems on the emotions experienced in adults and in 9-year-old children
Source: PLoS One. 2023 Nov 29;18(11):e0289027. doi: 10.1371/journal.pone.0289027 (PMC10686422; doi:10.1371/journal.pone.0289027)
Supplement: S1 Table — Percentages of explained variance for each component are presented in parentheses. (DOCX) [file pone.0289027.s003.docx]

# **Supplementary materials**

| **Table S1**  Strong component factor loading (≥ .7) for each regression for Epistemic Emotions (E) - Applicative Problems (AP) - Feedback (FB). Percentages of explained variance for each component are presented in parentheses | | | | | | |
| --- | --- | --- | --- | --- | --- | --- |
|  | Component 1  (24.49%) | Component 2  (17.80%) | Component 3  (15.03%) | Component 4  (12.19%) | | Component  5  (8.70%) |
| Joy | .915 |  |  | |  |  |
| Happiness | .873 |  |  | |  |  |
| Excitement | .847 |  |  | |  |  |
| Curiosity | .827 |  |  | |  |  |
| Interest | .776 |  |  | |  |  |
| Nervousness |  | .933 |  | |  |  |
| Anxiety |  | .821 |  | |  |  |
| Worry |  | .760 |  | |  |  |
| Frustration |  |  | .875 | |  |  |
| Irritation |  |  | .850 | |  |  |
| Dissatisfaction |  |  | .765 | |  |  |
| Astonishment |  |  |  | | .874 |  |
| Surprise |  |  |  | | .753 |  |
| Monotonous |  |  |  | |  | .868 |
