# Supplementary material for: Effects of two types of numerical problems on the emotions experienced in adults and in 9-year-old children
Source: PLoS One. 2023 Nov 29;18(11):e0289027. doi: 10.1371/journal.pone.0289027 (PMC10686422; doi:10.1371/journal.pone.0289027)
Supplement: S2 Table — Percentages of explained variance for each component are presented in parentheses. (DOCX) [file pone.0289027.s004.docx]

# **Supplementary materials**

**Table S2**

*Strong component factor loading (≥ .7) for each regression for Epistemic Emotions (E) - Applicative Problems (AP) - No Feedback (NFB). Percentages of explained variance for each component are presented in parentheses*

|  | Component 1  (21.20%) | Component 2  (32.88%) | Component 3  (12.30%) | Component 4  (8.48%) | |
| --- | --- | --- | --- | --- | --- |
| Joy | .792 |  |  | |  |
| Happiness | .780 |  |  | |  |
| Excitement | .840 |  |  | |  |
| Curiosity | .766 |  |  | |  |
| Interest | .856 |  |  | |  |
| Nervousness |  | .838 |  | |  |
| Anxiety |  | .875 |  | |  |
| Worry |  | .754 |  | |  |
| Frustration |  | .840 |  | |  |
| Irritation |  | .727 |  | |  |
| Dissatisfaction |  | .810 |  | |  |
| Confusion |  | .769 |  | |  |
| Astonishment |  |  | .766 | |  |
| Surprise |  |  | .863 | |  |
| Monotonous |  |  |  | | .859 |
