# Supplementary material for: Effects of two types of numerical problems on the emotions experienced in adults and in 9-year-old children
Source: PLoS One. 2023 Nov 29;18(11):e0289027. doi: 10.1371/journal.pone.0289027 (PMC10686422; doi:10.1371/journal.pone.0289027)
Supplement: S3 Table — Percentages of explained variance for each component are presented in parentheses. (DOCX) [file pone.0289027.s005.docx]

# **Supplementary materials**

**Table S3**

*Strong component factor loading (≥ .7) for each regression for Epistemic Emotions (E) – Non-Applicative Problems (NAP) - Feedback (FB). Percentages of explained variance for each component are presented in parentheses*

|  | Component 1  (22.98%) | Component 2  (14.94%) | Component 3  (25.30%) | Component 4  (12.74%) | |
| --- | --- | --- | --- | --- | --- |
| Joy | .948 |  |  |  |  |
| Happiness | .887 |  |  |  |  |
| Excitement | .927 |  |  |  |  |
| Curiosity | .799 |  |  |  |  |
| Interest | .724 |  |  |  |  |
| Nervousness |  | .787 |  |  |  |
| Anxiety |  | .875 |  |  |  |
| Worry |  | .856 |  |  |  |
| Frustration |  |  | .755 |  |  |
| Confusion |  |  | .788 |  |  |
| Perplexity |  |  | .797 |  |  |
| Astonishment |  |  | .820 |  |  |
| Surprise |  |  | .856 |  |  |
| Monotonous |  |  |  | .721 |  |
| Boredom |  |  |  | .844 |  |
