# Supplementary material for: Effects of two types of numerical problems on the emotions experienced in adults and in 9-year-old children
Source: PLoS One. 2023 Nov 29;18(11):e0289027. doi: 10.1371/journal.pone.0289027 (PMC10686422; doi:10.1371/journal.pone.0289027)
Supplement: S4 Table — Percentages of explained variance for each component are presented in parentheses. (DOCX) [file pone.0289027.s006.docx]

# **Supplementary materials**

**Table S4**

*Strong component factor loading (≥ .7) for each regression for Epistemic Emotions (E) – Non-Applicative Problems (NAP) - No Feedback (NFB). Percentages of explained variance for each component are presented in parentheses*

|  | Component 1  (22.42%) | Component 2  (20.94%) | Component 3  (21.77%) | Component 4  (10.07%) | |  |
| --- | --- | --- | --- | --- | --- | --- |
| Joy | .767 |  |  | |  | |
| Happiness | .823 |  |  | |  | |
| Excitement | .849 |  |  | |  | |
| Curiosity | .792 |  |  | |  | |
| Interest | .835 |  |  | |  | |
| Nervousness |  | .896 |  | |  | |
| Anxiety |  | .849 |  | |  | |
| Worry |  | .815 |  | |  | |
| Frustration |  |  | .742 | |  | |
| Irritation |  |  | .746 | |  | |
| Dissatisfaction |  |  | .730 | |  | |
| Perplexity |  |  | .803 | |  | |
| Astonishment |  |  |  | |  | |
| Surprise |  |  |  | |  | |
| Monotonous |  |  |  | | .809 | |
