# Supplementary material for: Effects of two types of numerical problems on the emotions experienced in adults and in 9-year-old children
Source: PLoS One. 2023 Nov 29;18(11):e0289027. doi: 10.1371/journal.pone.0289027 (PMC10686422; doi:10.1371/journal.pone.0289027)
Supplement: S5 Table — Percentages of explained variance for each component are presented in parentheses. (DOCX) [file pone.0289027.s007.docx]

# **Supplementary materials**

| **Table S5**  Strong component factor loading (≥ .7) for each regression for Achievement Emotions (A) - Applicative Problems (AP) - Feedback (FB). Percentages of explained variance for each component are presented in parentheses | | |
| --- | --- | --- |
|  | Component 1  (32.80%) | Component 2  (31.62%) |
|  |  |  |
| Relief | .777 |  |
| Pride | .818 |  |
| Joy | .743 |  |
| Optimism | .731 |  |
| Shame |  | .878 |
| Despair |  | .730 |
| Anger |  | .722 |
