# Supplementary material for: Effects of two types of numerical problems on the emotions experienced in adults and in 9-year-old children
Source: PLoS One. 2023 Nov 29;18(11):e0289027. doi: 10.1371/journal.pone.0289027 (PMC10686422; doi:10.1371/journal.pone.0289027)
Supplement: S6 Table — Percentages of explained variance for each component are presented in parentheses. (DOCX) [file pone.0289027.s008.docx]

# **Supplementary materials**

| **Table S6**  Strong component factor loading (≥ .7) for each regression for Achievement Emotions (A) - Applicative Problems (AP) - No Feedback (NFB). Percentages of explained variance for each component are presented in parentheses | | |
| --- | --- | --- |
|  | Component 1  (30.30%) | Component 2  (38.13%) |
| Relief | .705 |  |
| Pride | .827 |  |
| Joy | .771 |  |
| Optimism | .738 |  |
| Shame |  | .823 |
| Despair |  | .843 |
| Anger |  | .774 |
| Anxiety |  | .834 |
