# Supplementary material for: Effects of two types of numerical problems on the emotions experienced in adults and in 9-year-old children
Source: PLoS One. 2023 Nov 29;18(11):e0289027. doi: 10.1371/journal.pone.0289027 (PMC10686422; doi:10.1371/journal.pone.0289027)
Supplement: S7 Table — Percentages of explained variance for each component are presented in parentheses. (DOCX) [file pone.0289027.s009.docx]

# **Supplementary materials**

| **Table S7**  Strong component factor loading (≥ .7) for each regression for Achievement Emotions (A) – Non-Applicative Problems (NAP) - Feedback (FB). Percentages of explained variance for each component are presented in parentheses | | |
| --- | --- | --- |
|  | Component 1  (30.77%) | Component 2  (33.93%) |
| Relief | .755 |  |
| Pride | .700 |  |
| Joy | .888 |  |
| Optimism | .742 |  |
| Shame |  | .805 |
| Despair |  | .845 |
| Anger |  | .758 |
| Anxiety |  | .773 |
