# Supplementary material for: Effects of two types of numerical problems on the emotions experienced in adults and in 9-year-old children
Source: PLoS One. 2023 Nov 29;18(11):e0289027. doi: 10.1371/journal.pone.0289027 (PMC10686422; doi:10.1371/journal.pone.0289027)
Supplement: S8 Table — Percentages of explained variance for each component are presented in parentheses. (DOCX) [file pone.0289027.s010.docx]

# **Supplementary materials**

| **Table S8**  Strong component factor loading (≥ .7) for each regression for Achievement Emotions (A) – Non-Applicative Problems (NAP) – No Feedback (NFB). Percentages of explained variance for each component are presented in parentheses | | | |
| --- | --- | --- | --- |
|  | Component 1  (32.87%) | Component 2  (34.31%) | Component 3  (14.20%) |
| Relief |  |  | .952 |
| Pride | .881 |  |  |
| Joy | .881 |  |  |
| Optimism | .914 |  |  |
| Shame |  | .839 |  |
| Despair |  | .849 |  |
| Anger |  | .876 |  |
